# Symptom Burden in Patients on Maintenance Hemodialysis: Prevalence and Severity 17 Years Apart

**DOI:** 10.3390/jcm13185529

**Published:** 2024-09-18

**Authors:** Maurizio Bossola, Ilaria Mariani, Carlo Pasquale Piccinni, Claudia Spoliti, Enrico Di Stasio

**Affiliations:** 1 Servizio Emodialisi, Dipartimento di Scienze Mediche e Chirurgiche, Università Cattolica del Sacro Cuore, 00168 Rome, Italy; ilaria.mariani04@icatt.it (I.M.);; 2 Policlinico Universitario Fondazione Agostino Gemelli IRCCS, 00168 Rome, Italy; 3 Dipartimento di Medicina e Chirurgia Traslazionale, Università Cattolica del Sacro Cuore, 00168 Rome, Italy; 4 Dipartimento di Scienze Biotecnologiche di Base, Cliniche Intensivologiche e Perioperatorie, Università Cattolica del Sacro Cuore, 00168 Rome, Italy

**Keywords:** hemodialysis, symptom, symptom burden, prevalence, severity

## Abstract

**Objectives**: The aim of this study is to compare data from two cohorts separated by a 17-year interval. We assessed the prevalence and severity of symptoms with the “dialysis symptom index” in these two groups, recruited in 2007 and 2024, to determine how advancements in dialysis therapy have influenced the symptom burden’s prevalence and severity. **Methods**: End-stage renal diseases patients receiving maintenance hemodialysis three times a week in the hemodialysis unit of the university hospital were recruited between February and March 2007. In May 2024, in the same unit, another population sample was recruited and studied, as in 2007. The Dialysis Symptom Index (DSI) was administered to each patient, during the dialysis treatment. The DSI is made up of 30 questions, each of which addresses a specific physical or emotional symptom. The total symptom burden score, representing the total number of symptoms reported as being present, and the total symptom severity score, which represents the sum of individual severity scores, were generated for each patient. **Results**: We studied 71 patients in 2007 and 61 patients in 2024. The demographic, clinical and laboratory characteristics of the two study populations did not differ significantly. The total symptom burden score did not differ significantly between 2007 and 2024. The prevalence of most symptoms was similar in the two groups. The prevalence of constipation, decreased interest in sex and difficulty in becoming sexually aroused was higher in 2024 than in 2007. The total symptom severity was similar in the two periods. The severity of most symptoms was similar in the two groups. The severity of decreased interest in sex and difficulty in becoming sexually aroused was higher in 2024 than in 2007. **Conclusions**: The present study shows that, 17 years apart, the prevalence and severity of the symptom burden in patients on maintenance hemodialysis did not change significantly. These results suggest that studies investigating the causes and the pathogenesis of symptoms of patients on maintenance hemodialysis are urgently needed in the next future, as well as studies on therapeutic strategies.

## 1. Introduction

The global hemodialysis population is growing rapidly, and it has been estimated that in 2020, the number of people receiving hemodialysis exceeded 2.5 million, and that it will rise to 5.4 million by 2030 [1,2].

In the last 20 years, there has been an enormous improvement in hemodialysis treatments in terms of techniques, filters and intradialytic support (drugs for anemia secondary to kidney disfunction, such as erythropoietin; drugs for hyperparathyroidism, such as calcimimetics) [3,4]. Despite the many advances in hemodialysis technologies and patient access, patients on chronic hemodialysis still have a huge symptom burden along with functional and social problems that impact significantly their quality of life (QoL). The QoL of patients on maintenance hemodialysis is significantly lower than that of healthy individuals or of patients affected by other chronic diseases [5,6,7,8]. Interestingly, the symptom burden of patients on chronic hemodialysis is very heavy, and the severity of symptoms is generally moderate or high [9,10].

Patients on maintenance hemodialysis experience a multitude of physical (pain, fatigue, muscle cramps, anorexia, xerostomia, insomnia, headache, itching, taste change, numbness) and psychological (anxiety, depression, feeling nervous, feeling irritable) symptoms. The co-occurrence of symptoms in the same patient has led to the identification of symptom clusters in patients on maintenance hemodialysis [11,12,13,14].

Unfortunately, it is still not known whether the symptom burden in hemodialysis patients has improved along with the substantial research efforts and, above all, with the improvements in dialytic techniques and supportive therapies. Rather, it seems that the rapid expansion in the provision of dialysis was not followed by a patient-centered intervention.

The aim of this study is to compare data from two cohorts separated by a 17-year interval. We assessed the prevalence and severity of symptoms with the “dialysis symptom index” in these two groups, recruited in 2007 and 2024, to determine how advancements in dialysis therapy have influenced the symptom burden’s prevalence and severity.

## 2. Methods

### 2.1. Patient Population

End-stage renal diseases patients receiving maintenance hemodialysis three times a week in the hemodialysis unit of the university hospital were recruited between February and March 2007. In May 2024, in the same unit, another population sample was recruited and studied, as in 2007. This dialysis facility is staffed by academic nephrologists.

Exclusion criteria were age < 18 years, cognitive impairment. Informed consent was obtained from all subjects involved in the study. The study was conducted according to the guidelines of the Declaration of Helsinki, and approved by the Ethics Committee (P/606/CE2011).

### 2.2. Demographic and Clinical Data Collection

At the time of subject enrollment, in both periods, demographic (age, sex), clinical (dialytic age, Charlson comorbidity index, primary cause of end-stage renal disease) and laboratory (hemoglobin, serum creatinine, calcium, phosphorus, albumin, parathyroid hormone, Kt/V) variables were collected for each patient.

### 2.3. Assessment of Symptoms

The Italian version of the Dialysis Symptom Index (DSI) (15) was administered to each patient during the dialysis treatment, by the study coordinators. The DSI is made up of 30 questions, each of which addresses a specific physical or emotional symptom. Patients were asked to report symptoms that had been present at any time during the previous week by responding “yes” or “no”. For symptoms that were present, the patient was then asked to describe the symptom severity on a five-point Likert scale (1 = not bothersome to 5 = bothers very much). A total symptom burden score, which represents the total number of symptoms reported as being present, was generated, as well as a total symptom severity score, which represents the sum of individual severity scores [15].

### 2.4. Statistical Analyses

The Fisher’s exact test was used to compare the prevalence of individual symptoms and the total symptom burden scores between the two populations. The Mann–Whitney U test was used to compare individual and total symptom severity scores between the two patient groups. Similar statistical methods were used to compare demographic and clinical variables between the two populations. Statistical analysis was performed using SPSS software, version 25 (IBM, Armonk, NY, USA).

### 2.5. Type of Hemodialysis

Patients were receiving conventional 4 h bicarbonate hemodialysis (HD), three times a week. The dialysis treatment duration was 240 min. In HD, the blood flow ranged from 250 to 300 mL/min, with a dialysis rate flow of 500 mL/min. High-flux membranes were used.

## 3. Results

### 3.1. Patient Characteristics

We studied 71 patients in 2007 and 61 patients in 2024. As shown in Table 1, the demographic, clinical and laboratory characteristics of the two study populations did not differ significantly. The serum albumin levels were significantly higher in 2024 and the serum creatinine levels were significantly lower in 2024.

### 3.2. Symptom Prevalence

The total symptom burden score did not differ significantly between 2007 and 2024 (Table 2). As shown in Table 3, the prevalence of most symptoms was similar in the two groups. The prevalence of constipation, decreased interest in sex and difficulty in becoming sexually aroused was higher in 2024 than in 2007.

### 3.3. Symptom Severity

The total symptom severity was similar in the two periods (Table 2). As shown in Table 4, the severity of most symptoms was similar in the two groups. The severity of decreased interest in sex and difficulty in becoming sexually aroused was higher in 2024 than in 2007.

### 3.4. Symptom Burden and Variables Associated

In the 2007 cohort, we stratified the patients, according to the number of symptoms, into two groups (≤ e > the median (95% CI) value was 14 (11–16)). The two groups did not differ significantly for age (*p* = 0.165), sex (*p* = 0.488), dialysis vintage (*p* = 0.111), Charlson comorbidity index (*p* = 0.133), Kt/V (*p* = 0.282), serum albumin (*p* = 0.088), or PTH (*p* = 0.934). Similarly, in the 2024 cohort, we stratified the patients, according to the number of symptoms, into two groups (≤ e > the median (95% CI) value was 14 (13–15)). The two groups did not differ significantly for age (*p* = 0.545), sex (*p* = 0.062), dialysis vintage (0.901), Charlson comorbidity index (*p* = 0.856), Kt/V (*p* = 0.517), or serum albumin (*p* = 0.478), PTH (*p* = 0.913).

## 4. Discussion

The present observational study shows that the symptom burden’s prevalence and severity did not change significantly 17 years apart in patients on maintenance hemodialysis in the outpatient dialysis unit of a university hospital. The populations studied are representative of the hemodialysis population of the local region of our country [16].

In the past, it was reported that nephrologists generally were largely unaware of the presence of physical and emotional symptoms among patients on chronic hemodialysis, or that they underestimated their severity [16,17,18]. Historically, we always paid great attention to the screening and recognition of the physical and emotional symptoms of patients receiving maintenance hemodialysis in our hospital, and intense research activity has been dedicated, in these last years, to the diagnosis and management of physical and emotional symptoms in such patients [19,20,21,22,23,24]. Routine symptom assessment is conducted every year in our unit, although the optimal frequency of such assessment in dialysis patients to eventually improve outcomes without overburdening the patients is essentially unknown [24]. At the same time, in these last 17 years, we provided to the patients in our hemodialysis unit every technical and pharmacological armamentarium to improve their outcomes. Thus, considering that, in our experience, the policy of the screening and recognition of the physical and emotional symptoms did not translate into their improvement, it remains to understand and define the causes of this failure.

When compared to other chronic diseases, a lower number of studies on the treatment of physical and emotional symptoms has been conducted in patients on maintenance hemodialysis. For instance, since 1970, 1280 studies have been published about the treatment of fatigue in hemodialysis, 4737 on fatigue in multiple sclerosis, and 29,000 on cancer fatigue.

One reason for the paucity of such studies is the poor knowledge of the causes of the symptoms of patients on maintenance hemodialysis. The causes of insomnia remain essentially unknown, although it has been suggested that chronic inflammation, impaired or altered metabolism of sleep-regulatory mediators and sleep disruption related to treatment may be involved [25]. Similarly, the exact pathogenesis of uremic pruritus remains unknown, although it has been shown to be associated with increased systemic inflammation, abnormal serum parathyroid hormone, calcium and phosphorus levels, an imbalance in opiate receptors and a neuropathic process [26]. Despite being a high priority for patients, fatigue temporally associated with maintenance HD treatments is an under-investigated phenomenon among patients receiving hemodialysis [19]. It has been shown that fatigue in hemodialysis patients may be associated with inflammation, depression, dialytic age, or age, but the causes remain essentially unknown [19]. Xerostomia may be secondary to the use of some medications, but it seems to be due largely to a multifactorial mechanism, with the exact cause remaining unknown [20]. Little is known about the pathogenesis of uremia-related anorexia, considering that the hypothesis of a role played by uremic toxins, middle molecules, inflammation and altered amino acid pattern are not supported by consistent data [22].

In the absence of knowledge about the causes and pathogenesis of symptoms, treatments are scarce. In addition, therapies that are common in healthy individuals may not be indicated in patients on maintenance hemodialysis. This is the case, for instance, in the use of opioids, or in the use of non-steroidal anti-inflammatory agents for the treatment of pain [27]. Although depression is very common in patients on chronic hemodialysis [28], guidelines specific for this population are not available, so far. In clinical practice, it has been shown that the use of selective serotonin reuptake inhibitors (SSRIs) is common among hemodialysis patients who receive treatment for depression [28]. However, a recent systematic review has shown that further randomized, controlled studies are needed to determine whether SSRIs may be used routinely in daily clinical practice in such a population [29]. On insomnia, there is limited evidence on effective treatments for this population. A recent randomized study failed to demonstrate better efficacy for cognitive behavioral therapy or trazodone compared with placebo [30]. Accordingly, Lindner et al. have recently concluded that “limited intervention trials are available to establish an appropriate evidence base for specific treatment recommendations” [31,32]. With regard to fatigue, although cold dialysate, frequent dialysis, clearance of large middle molecules, treatment of depression and exercise seem useful, the limitations of the studies (lack of a control group, observational design, or short intervention duration) restrict their applicability in routine clinical practice [19]. The use of chewing-gum, mouthwash, acupressure, or transcutaneous electrical stimulation has led to conflicting and not definitive results in the treatment of xerostomia [20]. For the management of anorexia, the therapeutic armamentarium is very poor and no effective therapy is available, so far [22]. Dopaminergic drugs and calcium channel blockers have proven to be helpful for the treatment of restless legs syndrome, although high-quality studies with these agents are currently underway, and it is unknown whether their efficacy will be confirmed [33,34].

The present study also shows that all the demographic, clinical and laboratory variables were not associated with the symptom burden, either in the 2007 sample or in the 2024 one. These results must be interpreted with caution, considering the relatively small size of the patient groups and the limited number of demographic, clinical and laboratory variables included in the analysis. In fact, a recent meta-analysis demonstrated that the symptom burden of patients on maintenance hemodialysis was positively correlated with age, gender, working status, medical cost, dialysis vintage, quality of sleep, nutritional status, comorbidities, depression, anxiety, avoidance coping and resignation coping, and negatively correlated with marital status, income, serum sodium, quality of life, social support, subjective well-being, and self-management ability.

In conclusion, the present study shows that, 17 years apart, the prevalence and severity of the symptom burden in patients on maintenance hemodialysis did not change significantly. These results suggest that effort should be made to design adequate studies on the causes and pathogenesis of physical and emotional symptoms in patients on chronic hemodialysis and, once the underlying mechanisms are identified, it is desirable for high-quality studies on the possible therapeutic pharmacological and non-pharmacological interventions to be performed.

## Figures and Tables

**Table 1:** Characteristics of patients in 2007 and in 2024.

	Group 1 (n = 71)	Group 2 (n = 61)	*p*
Age (years)	63 ± 15 65 (25–89)	64 ± 13 65 (31–84)	0.866
Sex (male/female)	42:29	34:27	0.726
Dialysis vintage (years)	5 (4.1–5.6)	4.7 (3.7–5.5)	0.792
Primary cause of ESRD:			
hypertension	20 (28.1%)	19 (31.4%)	
glomerulonephritis	16 (22.5%)	14 (22.9%)	
diabetes mellitus	19 (26.7%)	17 (27.8%)	
interstitial nephritis	7 (9.8%)	5 (8.2%)	
polycystic kidney disease	5 (7%)	4 (6.5%)	
others/unknown	4 (5.6%)	2 (3.3%)	0.986
Charlson Index	3 (2–7)	2 (2–6)	0.134
Kt/V	1.33 ± 0.16	1.34 ± 0.25	0.781
Hemoglobin (g/dL)	11 ± 1 11 (8–14)	11 ± 1 11 (8–13)	0.686
Serum albumin (g/L)	35.1 ± 4.9 36 (21–43)	39.4 ± 2.4 39 (34–44)	<0.0001
Serum creatinine (mg/dL)	10.3 ± 2.9 9.7 (4.4–18.9)	8.1 ± 1.5 8.3 (5.6–12.3)	<0.0001
Calcium (mg/dL)	9.2 ± 0.4	9.1 ± 0.6	0.256
Phosphorus (mg/dL)	5.6 ± 2.2	5.4 ± 1.54	0.549
PTH (pg/mL)	301 (135–286)	285 (151–310)	0.827

Data are presented as mean ± SD or median (95% CI). ESRD, end-stage renal disease; PTH, parathyroid hormone.

**Table 2:** Total symptom burden score and total symptom severity score in 2007 and in 2024.

	Group 1 (n = 71)	Group 2 (n = 61)	*p*
Total symptom burden score (the total number of symptoms reported as being present)	13.3 ± 6.1	13.4 ± 4.5	0.903
Total symptom severity score (the sum of individual severity scores)	23.5 (20–26)	27 (22.3–31)	0.215

Data are presented as mean ± SD or median (95% CI).

**Table 3:** Symptoms’ prevalence in 2007 and in 2024.

	Group 1 (n = 71)	Group 2 (n = 61)	*p*
Constipation	18 (25.3%)	27 (44.2%)	0.027
Nausea	17 (23.9%)	13 (21.3%)	0.835
Vomiting	7 (9.8%)	12 (19.7%)	0.137
Diarrhea	19 (26.7%)	16 (26.2%)	0.556
Decreased appetite	27 (38%)	24 (39.3%)	1.000
Muscle cramps	44 (62%)	32 (52.4%)	0.293
Swelling in legs	15 (21.1%)	11 (18%)	0.826
Shortness of breath	28 (39.4)	26 (42.6%)	0.855
Dizziness	21 (29.5%)	15 (24.5%)	0.561
Restless legs	31 (43.6%)	17 (27.9%)	0.070
Numbness or tingling in feet	26 (36.6%)	19 (31.1%)	0.582
Feeling tired or lack of energy	63 (88.7%)	50 (82%)	0.324
Cough	13 (18.3%)	17 (27.9%)	0.215
Dry mouth	38 (53.5%)	39 (63.9%)	0.288
Bone or joint pain	46 (64.7%)	39 (63.9%)	1.000
Chest pain	8 (11.2%)	4 (6.5%)	0.382
Headache	15 (21.1%)	20 (32.8%)	0.166
Muscle soreness	41 (57.7%)	29 (47.5%)	0.294
Difficulty concentrating	29 (40.8%)	19 (31.1%)	0.279
Dry skin	50 (70.4%)	38 (62.3%)	1.000
Itching	44 (62%)	28 (45.9%)	0.080
Worrying	38 (53.5%)	42 (68.8%)	0.077
Feeling nervous	36 (50.7%)	34 (55.7%)	0.602
Trouble falling asleep	34 (47.8%)	26 (42.6%)	0.600
Trouble staying asleep	43 (60.5%)	29 (47.5%)	0.161
Feeling irritable	31 (43.6%)	34 (55.7%)	0.294
Feeling sad	41 (57.7%)	32 (52.4%)	0.600
Feeling anxious	37 (52.1%)	30 (49.2%)	0.861
Decreased interest in sex *	44 (64.7%)	46 (82.1%)	0.042
Difficulty in becoming sexually aroused *	42 (61.7%)	46 (82.1%)	0.016

* Three patients in 2007 and five patients in 2024 did not report data about these symptoms.

**Table 4:** Symptoms’ severity in 2007 and in 2024.

	Group 1 (n = 71)	Group 2 (n = 61)	*p*
Constipation	1 ± 1 0 (0–4)	1 ± 1 0 (0–4)	0.017
Nausea	0 ± 1 0 (0–4)	0 ± 1 0 (0–3)	0.669
Vomiting	0 ± 1 0 (0–3)	0 ± 1 0 (0–3)	0.130
Diarrhea	0 ± 1 0 (0–3)	0 ± 1 0 (0–2)	0.873
Decreased appetite	1 ± 1 0 (0–4)	1 ± 1 0 (0–4)	0.844
Muscle cramps	1 ± 1 1 (0–4)	1 ± 1 1 (0–3)	0.085
Swelling in legs	0 ± 1 0 (0–4)	0 ± 1 0 (0–3)	0.661
Shortness of breath	1 ± 1 0 (0–4)	1 ± 1 0 (0–3)	0.678
Dizziness	1 ± 1 0 (0–4)	0 ± 1 0 (0–3)	0.488
Restless legs	1 ± 1 0 (0–4)	1 ± 1 0 (0–4)	0.039
Numbness or tingling in feet	1 ± 1 0 (0–4)	1 ± 1 0 (0–3)	0.566
Feeling tired or lack of energy	2 ± 1 2 (0–4)	2 ± 1 2 (0–4)	0.938
Cough	0 ± 1 0 (0–3)	1 ± 1 0 (0–3)	0.147
Dry mouth	1 ± 1 1 (0–4)	1 ± 1 1 (0–4)	0.289
Bone or joint pain	1 ± 1 1 (0–4)	2 ± 2 1 (0–4)	0.433
Chest pain	0 ± 0 0 (0–2)	0 ± 0 0 (0–1)	0.362
Headache	0 ± 1 0 (0–3)	1 ± 1 0 (0–4)	0.121
Muscle soreness	1 ± 1 1 (0–4)	1 ± 1 0 (0–3)	0.103
Difficulty concentrating	1 ± 1 0 (0–3)	1 ± 1 0 (0–3)	0.273
Dry skin	1 ± 1 1 (0–4)	1 ± 1 1 (0–4)	0.444
Itching	1 ± 1 1 (0–4)	1 ± 1 0 (0–4)	0.057
Worrying	1 ± 1 1 (0–4)	1 ± 1 1 (0–4)	0.352
Feeling nervous	1 ± 1 1 (0–4)	1 ± 2 1 (0–12)	0.563
Trouble falling asleep	1 ± 1 0 (0–4)	1 ± 2 0 (0–13)	0.974
Trouble staying asleep	1 ± 1 1 (0–4)	1 ± 1 0 (0–4)	0.967
Feeling irritable	1 ± 1 0 (0–4)	1 ± 1 1 (0–4)	0.337
Feeling sad	1 ± 1 1 (0–4)	1 ± 1 1 (0–4)	0.925
Feeling anxious	1 ± 1 1 (0–4)	1 ± 1 0 (0–4)	0.969
Decreased interest in sex	2 ± 2 2 (0–4)	3 ± 2 3 (0–4)	0.004
Difficulty in becoming sexually aroused	2 ± 2 2 (0–4)	3 ± 2 3 (0–4)	0.003

Data are presented as mean ± SD or median (95% CI).

## Data Availability

Dataset available on request from the authors.
